# Viewpoint survey of mental health service users’ experiences of discrimination in England 2008–2012

**DOI:** 10.1007/s00127-014-0875-3

**Published:** 2014-07-20

**Authors:** R. C. Henderson, E. Corker, S. Hamilton, P. Williams, V. Pinfold, D. Rose, M. Webber, S. Evans-Lacko, G. Thornicroft

**Affiliations:** 1 Health Service and Population Research Department, David Goldberg Centre P029, King’s College London, Institute of Psychiatry, De Crespigny Park, London, SE5 8AF UK; 2 The McPin Foundation, 32-36 Loman Street, London, SE1 0EH UK; 3 International Centre for Mental Health Social Research, Department of Social Policy and Social Work, University of York, Heslington, York, YO10 5DD UK

**Keywords:** Mental health discrimination, Social capital, Mental health service users, Stigma, Welfare benefits

## Abstract

**Purpose:**

Research suggests levels of discrimination among mental health service users in England are high, but fell over the course of the first phase of the Time to Change programme to reduce stigma and discrimination (2008–2011). The aim of this study was to determine changes in discrimination levels, both overall and by the area of life in which discrimination is experienced, since Time to Change began and over the first year of its second phase (2011–2012).

**Method:**

Separate samples of mental health service users were interviewed annually from 2008 to 2012 using the Discrimination and Stigma Scale. In 2011 and 2012, social capital was also measured using the Resource Generator-UK.

**Results:**

Sample percentages of participants reporting the experience of discrimination in one or more life areas for years 2008–2012 were 91.4, 86.5, 86.2, 87.9 and 91.0 %, respectively. A multivariable logistic regression model was performed to test for significant differences by study year, weighted to match the study population and adjusted for employment status and diagnosis as potential confounding factors. The odds of reporting discrimination in one or more life areas were significantly lower as compared to 2008 for all subsequent years except for 2012 (0.76, 95 % CI 0.49–1.19). However, a weighted multiple regression model provided evidence of decreased mean overall discrimination in 2012 as compared to 2008 (mean decrease −7.57, 95 % CI −11.1 to −4.0, *p* < 0.001). The weighted mean number of social resources was 13.5 in 2012 as compared to 14.0 in 2011 (mean difference −0.60, 95 % CI −1.25 to 0.06).

**Conclusions:**

While the overall level of discrimination across the life areas studied has fallen over 2008–2012, there is no evidence that more people using mental health services experience no discrimination. We suggest that the pattern suggesting a recent rise in discrimination following an earlier reduction may be linked to economic austerity. Further, the welfare benefits system has become an increasing source of discriminatory experience.

## Introduction

Stigma and discrimination can significantly compound the difficulties facing people with mental health problems [1–3]. In England, despite greater understanding about the causes of these problems [4] public attitudes towards people with mental health problems may have deteriorated prior to 2008 [4]; a similar picture has emerged in the USA [5].

Between 2008 and 2011, the charities Mind and Rethink Mental Illness ran the first phase of Time to Change (TTC––see http://www.time-to-change.org.uk/), the largest ever programme in England to reduce stigma and discrimination against people with mental health problems. TTC subsequently secured funding from the Department of Health in England and Comic Relief for a second phase, 2011–2015. The evaluation of TTC was the first to measure discriminatory behaviour nationally, as rated by service users using an annual ‘Viewpoint’ survey [6, 7]. The Department of Health in England uses this measure as an indicator to monitor the effect of one of the six policy objectives of ‘No Health Without Mental Health’, namely ‘fewer people will experience stigma and discrimination [8, 9]. Over the first phase of TTC, there was a significant reduction in overall levels of discrimination, including significant reductions in discrimination from friends, family members and in social life generally [6]. However, in other areas of life such as health professionals and welfare benefits there were no improvements. Further, some early reductions over 2008–2009 [7] were not maintained, e.g. with respect to finding a job, and the reduction in overall discrimination did not increase after 2010 [6]. The purpose of this study was to determine changes in discrimination levels, overall and by the area of life in which discrimination is experienced, throughout the duration of TTC so far (2008–2012) and over the first year of its second phase (2011–2012). We also wished to assess changes over the first year of TTC Phase 2 in access to social capital, using a measure added to the survey in 2011.

Social capital is a multi-dimensional construct encompassing elements such as trust [10], social norms and reciprocity [11], features of social structures and networks [12] and the resources embedded within them [13]. Measures of social capital reflect different conceptualisations and theoretical traditions. Epidemiological studies appear most influenced by Putnam’s [11] conception of social capital [14], whereas social network approaches more clearly align the concept with recovery discourses [15]. Here, social capital is understood as resources within social networks, which are accessible to individuals through trusting and reciprocal relationships. Our hypothesis was that any change in access to social capital would be in the opposite direction to a change in experienced discrimination, based on theoretical and empirical grounds [16].

## Methods

### Design

Telephone interview surveys were carried out annually between 2008 (baseline) and 2012. Different samples were used each year. Participants were recruited through National Health Service (NHS) Mental Health trusts (service provider organisations). Participants were eligible to take part if they were aged 18–65, had any mental health diagnosis (excluding dementia), and had been in recent receipt of specialist mental health services (contact in the previous 6 months). Participants were excluded if they were not currently living in the community (e.g. in prison or hospital) since patients need to be available to take part in a sensitive, confidential telephone survey. Our target sample was 1,000 individual interviews in each year, based on power calculations to detect a 5 % change in discrimination experiences.

### Setting

Each year, five NHS mental health trusts across England were selected to take part. Trusts were intended to be representative of all such trusts in the country, based on the socioeconomic deprivation level of their catchment area. Catchment areas for the whole of England were ordered using a score calculated from census variables chosen on the basis of an established association with mental illness rates [17], including lack of access to a car, permanent sickness, unemployment, being single, divorced or widowed, and living in housing that is not self-contained. We then selected five trusts to ensure that areas in each quintile of socioeconomic deprivation were included. Different trusts and/or different regions within the same trusts were selected each year.

### Participants

Within each participating trust, non-clinical staff in information technology or patient records departments used their central patient database to select a random sample of persons receiving care for ongoing mental health problems. In 2008, we invited 2,000 outpatients per trust based on a predicted response rate of 25 % as achieved for the charity Rethink Mental Illness membership surveys. In 2009–2012, it was 4,000 outpatients per trust to ensure we met the target sample after missing this in 2008. The sample was checked by clinical care teams to confirm eligibility and remove those who were judged to be at risk of distress from receiving an invitation to participate.

Invitation packs were mailed to potential participants from the trusts (8,917 in 2008; 12,887 in 2009; 12,866 in 2010; 9,120 in 2011 and 9,894 in 2012). They contained complete information about the study including lists of interview topics, local and national sources of support, and a consent form. After 2008, information was also included in 13 commonly spoken languages explaining how to obtain the information pack in another language if needed. A reminder letter was mailed to non-responders after ~2 weeks. Participants mailed written consent forms, including contact details, directly to the research team. Participants in 2011–2012 were offered a £10 voucher for taking part in the survey.

All telephone interviewers were trained and supervised by the research team. The majority of interviewers were mental health service users. Participants were allocated to interviewers according to availability. Once an interviewer made contact with a participant, an interview was conducted or scheduled. If, after three scheduled appointments, an interview had not been successfully completed, the participant was considered to have withdrawn. Consent was confirmed verbally by the interviewer prior to start of the interview.

### Measures

The Discrimination and Stigma Scale (DISC) was used to measure experienced discrimination and anticipated discrimination [18]. The DISC is interviewer administered, in this case by telephone, and contains: 22 items on negative, mental health-related experiences of discrimination, covering 21 specific life areas, plus one for ‘other’ experience; and four items concerning anticipated discrimination. All responses are given on a four point scale from ‘not at all’ to ‘a lot’. Where items related to situations which were not relevant to the participant in the previous 12 months (e.g. in relation to having children or seeking employment), or if a diagnosis could not have been known about in that situation, a ‘not applicable’ option was used. Recent analysis of the DISC has found that it has adequate psychometric properties [18].

The Resource Generator-UK (RG-UK) [19] was used to measure participants’ access to social capital in 2011–2012 as part of the evaluation of Phase 2 of Time to Change. In the tradition of social network measures such as the Name Generator [20] and Position Generator [21], this instrument measures participants’ access to social resources within their own social network. The RG-UK was derived from a version developed in The Netherlands [22] and its items have been made culturally relevant and validated for use in the UK general population. It has good reliability and validity [19] and has been used in samples of people with mental health problems, e.g. [16, 19, 23, 24] and produced valid findings.

The RG-UK asks participants whether or not they could obtain access to 27 skills and resources within their social network within 1 week if they needed it. If they respond ‘yes’ to an item, they are asked to indicate the nature of the social tie––i.e. close family, wider family, friends, colleague, acquaintance, mental health professional––through which they could access each skill or resource. The instrument has four subscales each representing a concrete domain of social capital to which an individual may have access: domestic resources, personal skills, expert advice and problem-solving resources. Participants were also asked whether they personally possessed 14 of the resources/skills. This accounts for the fact that people in possession of a skill or resource would be less likely to ask someone else with that skill or resource for their help. The mean score in a general population sample has been found to be 17.24 [19], providing a benchmark for other samples. Socio-demographic and clinical characteristics were also obtained from the sample.

### Statistical analysis

Analysis used STATA (version 13). Overall experienced discrimination scores were calculated by counting any reported instance of negative discrimination as ‘1’ and situations in which no discrimination was reported as ‘0’. The overall score was then calculated as: sum of reported discrimination divided by the number of questions answered (only applicable answers were included) and multiplied by 100. This gave a percentage of items in which discrimination was reported. For example, if a participant reported discrimination for 13 out of the possible 22 items and also reported that 4 items were not applicable, then the overall score would be 13/(22–4) × 100 = 72 %. To compare the 2008–2011 samples for frequencies of experiences from each source of discrimination (i.e. each DISC item), a binary variable––‘no discrimination’ versus ‘any discrimination’ was created for each DISC item, and ‘not applicable’ responses were coded as ‘no discrimination’. In 2008, three items were used to measure anticipated discrimination. One was split into two items from 2009; we therefore only compared the two items common to all years. Items were compared using a binary variable, no anticipated discrimination versus any anticipated discrimination, controlling for differences in demographics.

RG-UK total and subscale scores were calculated by scoring items accessible within a participant’s network as 1 and those not accessible as 0, and then summing to calculate scale totals.

Each yearly sample was compared to population level data made available by the NHS Information Centre [19] for characteristics on which good NHS data were available, i.e. gender, age and ethnicity. Inverse probability weights were then created based on these characteristics to weight observations for demographic disparities between the sample and the population, defined as the population of individuals aged 18–65 residing within the UK and accessing inpatient or outpatient mental health services.

Chi-squared tests were carried out to check for differences in socio-demographic characteristics between the years. Significant differences were found for ethnicity, employment status and diagnosis between years, but not for gender or age. Analyses were, therefore, also adjusted for ethnicity, employment status and diagnosis. The Bonferroni correction method was used to maintain the familywise type I error at 0.05 due to multiple testing for the life area items.

The study received approval from Riverside NHS Ethics Committee 07/H0706/72.

## Results

We interviewed a total of 4,583 participants between 2008 and 2012. For details of participant characteristics, see Table 1. Response rates in 2008, 2009, 2010, 2011 and 2012 were 6, 7, 7.6, 10.8 and 10.3 %, respectively. The increase in response rate in 2011 followed the introduction of a £10 token as compensation for participation. In all years, women and White British participants were over-represented in our sample compared with data available from the NHS Information Centre [25].

**Table 1:** Demographic characteristics of participants

Characteristic	2008 (*n* = 537)	2009 (*n* = 1,047)	2010 (*n* = 979)	2011 (*n* = 1,016)	2012 (*n* = 1,004)
Gender, *n* (%)					
Male	188 (35)	389 (37)	369 (38)	411 (40.5)	387 (38.5)
Female	344 (64)	654 (63)	605 (62)	602 (59.3)	617 (61.5)
Transgender	0 (0)	3 (0.3)	5 (0.5)	2 (0.2)	0 (0)
Age (years), mean (SD)	46 (11)	46 (11)	46 (11)	45 (11)	44 (11)
Ethnicity, *n* (%)					
White	515 (98)	955 (92)	918 (94)	904 (90)	898 (90)
Non-white	11 (2)	81 (8)	57 (6)	105 (10)	101 (10)
Employment status, *n* (%)					
Employed	147 (27)	282 (27)	298 (24)	239 (24)	222 (22)
Studying/training/volunteering/other	56 (10)	305 (29)	224 (23)	196 (19)	238 (24)
Unemployed	264 (49)	355 (34)	370 (38)	485 (48)	478 (48)
Retired	70 (13)	104 (10)	85 (9)	95 (9)	64 (6)
Clinical diagnosis, *n* (%)					
Anxiety disorders	36 (8)	59 (6)	57 (6)	832 (9)	86 (9)
Bipolar disorder	147 (32)	257 (26)	194 (21)	184 (20)	218 (24)
Depression	137 (29)	291 (30)	331 (36)	331 (34)	257 (28)
Personality disorders	20 (4)	60 (6)	41 (5)	55 (6)	71 (8)
Schizophrenia and schizoaffective disorder	75 (16)	169 (17)	137 (15)	142 (16)	200 (22)
Other	51 (11)	142 (15)	147 (16)	131 (14)	85 (9)
Received involuntary treatment, *n* (%)					
Yes	212 (40)	418 (40)	309 (32)	353 (35)	424 (42.4)
No	325 (60)	628 (60)	668 (68)	663 (65)	577 (57.5)

### Experienced discrimination

Sample percentages of participants reporting one or more experiences of discrimination for years 2008–2012 were 91.4, 86.5, 86.2, 87.9 and 91.0 %, respectively. Percentages weighted to match the demographics of the population in terms of gender, age and ethnicity were 92.3, 86.2, 87.2, 88.8 and 90.8 % for the same period. A multivariable logistic regression model for report of one or more experiences of discrimination was performed to test for significant differences by study year, weighted to match the study population in terms of gender, age and ethnicity. The model included employment status and diagnosis as potential confounding factors as these sample characteristics were found to differ between the cross-sectional samples each year. No evidence was found for a difference in 2012 as compared to 2008 (OR 0.76, 95 % CI 0.49–1.19, *p* = 0.230) or compared to 2011 (OR 1.26, 95 % CI 0.89–1.79, *p* = 0.199). In contrast, there was evidence for reduction in experienced discrimination in one or more life areas comparing each of 2009 (OR 0.50, 95 % CI 0.32–0.76, *p* = 0.001); 2010 (OR 0.53, 95 % CI 0.34–0.83, *p* = 0.006); and 2011 (OR 0.60, 95 % CI 0.38–0.95, *p* = 0.029) with 2008.

The sample mean numbers of life areas in which participants reported discrimination for years 2008–2012 were 5.4, 4.3, 4.1, 4.7 and 5.3, respectively. Weighted means to match the population characteristics in terms of gender, age and ethnicity were 5.7, 4.3, 4.4, 4.9 and 5.4, respectively. A weighted multiple regression model adjusted for employment status and diagnosis provided evidence of increased mean number of life areas in 2012 as compared to 2009 (mean increase 0.97, 95 % CI 0.62–1.32, *p* < 0.001), compared to 2010 (mean increase 1.02, 95 % CI 0.59–1.44, *p* < 0.001) and compared to 2011 (mean increase 0.48, 95 % CI 0.08–0.89, *p* = 0.019). These differences equated to standardised effect sizes of 0.27, 0.28 and 0.13, respectively. No evidence was found for a change in 2012 as compared to 2008 (mean decrease −0.35, 95 % CI −0.86 to 0.17, *p* = 0.186).

**Fig. 1:**
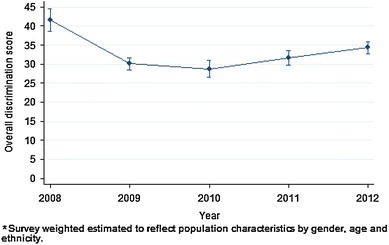
Overall discrimination score by year (weighted* estimates with 95 % CIs)

Figure 1 shows the overall discrimination score for each sample. The mean overall discrimination scores for each year of the study period were 40.3, 30.2, 28.0, 30.9 and 34.2 %, respectively. Weighted percentages for the years were 41.6, 30.1, 28.7, 31.7 and 34.3 %. A weighted multiple regression model (see Table 2) adjusted for employment status and diagnosis provided evidence of decreased mean overall discrimination in 2012 as compared to 2008 (mean decrease −7.57, 95 % CI −11.1 to −4.0, *p* < 0.001). The model also provided evidence of increased mean overall discrimination in 2012 as compared to 2009 (mean increase 3.5, 95 % CI 1.33–5.83, *p* = 0.002), 2010 (mean increase 5.23, 95 % CI 2.61–7.84, *p* < 0.001) and 2011 (mean increase 2.73, 95 % CI 0.26–5.19, *p* = 0.030). The above differences equated to standardised effect sizes of −0.32, +0.15, +0.22 and +0.11, respectively.

**Table 2:** Experienced discrimination, anticipated discrimination and social capital: 2008–2012 and 2011–2012

	Weighted descriptives^a^			2008–2012**				2011–2012**			
	2008	2011	2012	Estimate	95 % CI	*p*	Standardised effect size	Estimate	95 % CI	*p*	Standardised effect size
Experienced discrimination^a^											
Any vs. none^†^	92.3 %	88.8 %	90.8 %	0.76	0.49 to 1.19	0.230	n/a	1.26	0.89 to 1.79	0.199	n/a
Number of life areas	5.7	4.9	5.4	−0.35	−0.86 to 0.17	0.186	–0.10	0.48	0.08 to 0.89	0.019	0.13
Overall discrimination score	41.6	31.7	34.3	−7.57	−11.13 to −4.01	0.000	−0.32	2.73	0.26 to 5.19	0.030	0.11
Anticipated discrimination											
Concealed MH from others (yes vs. no)†	72.9 %	70.5 %	77.0 %	1.40	0.94 to 2.09	0.099	n/a	1.48	1.15 to 1.90	0.002	n/a
Stopped from having close personal relationship (yes vs no)^†^	49.2 %	50.4 %	53.1 %	1.18	0.85 to 1.64	0.324	n/a	1.05	0.85 to 1.31	0.638	n/a
Social capital											
RG-UK total score	n/a	14.0	13.5	n/a				−0.60	−1.25 to 0.06	0.073	−0.10
RG-UK factor: domestic	n/a	4.0	3.9	n/a				−0.12	−0.33 to 0.09	0.275	−0.06
RG-UK factor: expert	n/a	4.2	4.1	n/a				−0.19	−0.45 to 0.07	0.144	−0.08
RG-UK factor: skills	n/a	2.8	2.6	n/a				−0.17	0.34 to −0.01	0.040	−0.11
RG-UK factor: problem solving	n/a	3.0	2.8	n/a				−0.10	−0.23 to 0.02	0.114	−0.08

** Results from multivariable linear regressions models with the exception of^ †^ where logistic models were used. Reported estimates are in the form of mean differences in outcome between years for the linear regression models, and odds ratios for the logistic models. All models were also adjusted for ethnicity, employment status and diagnosis as these were found to differ between years. The social capital models were also adjusted by age as this was found to differ between 2011 and 2012 samples. Inverse probability weights were used to reflect the population characteristics by gender, age and ethnicity

^a^Means and proportions were estimated on the weighted sample to reflect the population characteristics by gender, age and ethnicity

Table 3 shows the proportions of reported negative discrimination in 2008, 2011 and 2012 for the life areas covered by the DISC. Proportions were weighted to match the population in terms of gender, age and ethnicity. Across all years, the most common reports of discrimination came from: family, friends and social life contacts, or from a general report of being avoided or shunned. Comparing 2012 with 2008, there has been a significant increase in experienced discrimination with respect to claiming welfare benefits, and no other significant changes.

**Table 3:** Negative discrimination 2008–2012 and 2011–2012

	Proportion reporting discrimination^a^			2008–2012**				2011–2012**			
Life area	2008	2011	2012	OR	95 % CI	*p* value	Significant after Bonferroni correction	OR	95 % CI	*p* value	Significant after Bonferroni correction
Being shunned	0.07	0.11	0.14	1.12	0.82–1.55	0.477	NS	1.55	1.24–1.93	<0.001	
Friends	0.07	0.09	0.12	0.70	0.54–0.93	0.013	NS	1.52	1.22–1.89	<0.001	
Family	0.06	0.09	0.11	0.68	0.52–0.90	0.007	NS	1.20	0.97–1.50	0.092	NS
Social life	0.05	0.07	0.10	0.84	0.62–1.15	0.285	NS	1.47	1.17–1.84	0.001	
Mental health staff	0.04	0.07	0.08	0.82	0.61–1.11	0.208	NS	1.11	0.88–1.39	0.397	NS
Dating	0.04	0.05	0.06	0.77	0.55–1.06	0.105	NS	1.01	0.78–1.29	0.963	NS
Physical health	0.03	0.06	0.07	1.09	0.79–1.51	0.607	NS	1.14	0.90–1.45	0.267	NS
Neighbours	0.03	0.06	0.06	1.04	0.75–1.45	0.822	NS	1.00	0.77–1.29	0.984	NS
Find job	0.03	0.05	0.05	0.69	0.48–0.98	0.038	NS	1.02	0.78–1.34	0.888	NS
Privacy	0.03	0.05	0.05	0.76	0.53–1.10	0.147	NS	1.12	0.86–1.46	0.403	NS
Safety	0.03	0.05	0.06	1.13	0.77–1.65	0.532	NS	1.10	0.85–1.41	0.466	NS
Benefits	0.02	0.06	0.07	1.86	1.29–2.67	0.001		1.16	0.90–1.49	0.252	NS
Parenting	0.02	0.04	0.04	0.89	0.61–1.29	0.531	NS	1.05	0.79–1.40	0.714	NS
Keep job	0.02	0.04	0.04	0.79	0.52–1.20	0.264	NS	1.10	0.82–1.47	0.518	NS
Police	0.02	0.04	0.04	0.99	0.65–1.50	0.961	NS	0.97	0.73–1.30	0.859	NS
Housing	0.02	0.03	0.04	1.37	0.92–2.04	0.118	NS	1.33	0.98–1.82	0.067	NS
Education	0.02	0.03	0.03	0.82	0.49–1.39	0.468	NS	0.98	0.69–1.40	0.928	NS
Marriage	0.01	0.04	0.03	1.16	0.74–1.82	0.527	NS	0.84	0.62–1.13	0.248	NS
Transport	0.01	0.03	0.03	1.43	0.92–2.23	0.111	NS	1.14	0.82–1.58	0.432	NS
Starting a family	0.01	0.02	0.02	0.69	0.42–1.13	0.142	NS	0.94	0.63–1.41	0.775	NS
Religious activities	0.01	0.01	0.01	0.54	0.31–0.94	0.029	NS	1.27	0.76–2.13	0.369	NS

** Results from multivariable logistic regressions of report of discrimination on year, adjusted for ethnicity, employment status and diagnosis as these were found to differ between years. Inverse probability weights were used to reflect the population characteristics by gender, age and ethnicity

^a^Proportions were estimated on the weighted sample to reflect the population characteristics by gender, age and ethnicity

Comparing 2012 with 2011, experienced discrimination was reported more frequently for 20 of the 21 life areas; after allowing for multiple testing the changes were statistically significant for discrimination from friends, in social life, and the experience of being shunned. The increase in experienced discrimination with respect to welfare benefits was no longer significant after adjusting for employment status.

### Awareness of anti-stigma campaign and reported discrimination

From 2009 onwards, participants were asked whether they were aware of the Time to Change programme and whether they had participated in any of its activities. Using data from all relevant years, we compared discrimination scores of those participants who were aware of TTC (*n* = 273, median discrimination score 30.0, SD 22.9) and those who were not (*n* = 3,773, median discrimination score 27.3, SD 23.2). A Mann–Whitney test showed no significant difference between the groups’ overall discrimination scores, *p* = 0.35.

### Anticipated discrimination

Weighted percentages of participants reported as concealing their diagnosis in 2008, 2011 and 2012 were 72.9, 70.5, and 77.0 %, respectively. A logistic regression controlling for demographic differences shows evidence for a significant increase between 2011 and 2012 for feeling the need to conceal one’s diagnosis (OR 1.48, 95 % CI 1.15–1.90, *p* = 0.002), but not between 2008 and 2012, (OR 1.40, 95 % CI 0.94–2.09, *p* = 0.099). No differences between samples in stopping oneself from starting relationships were found in 2012 as compared to 2008 (OR 1.18, 95 % CI 0.85–1.64, *p* = 0.324) and 2011 (OR 1.05, 95 % CI 0.85–1.31, *p* = 0.638).

### Social capital

The weighted mean number of resources participants had access to was 14.0 in 2012 compared to 13.5 in 2011; the mean number of resources accessible to participants was significantly lower than the general population benchmark of 17.24 [19] in both 2011 (*p* < 0.001) and 2012 (*p* < 0.001).

The results show a small difference between 2011 and 2012 on the Skills subscale of the RG-UK. The mean difference between years was 0.18 resources lower in 2012 as compared to 2011 (95 % CI −0.35 to −0.02) and this represented a small effect (standardised effect size 0.11). This difference remained significant in sensitivity models adjusting for potential confounders between years. A difference was also found on the Problem-solving subscale. The mean difference was 0.15 resources lower in 2012 (95 % CI −0.28 to −0.02), but this difference disappeared in the sensitivity models adjusting for confounders. No other differences were found between years (RG-UK Total Score, Domestic subscale or Expert subscale).

## Discussion

### Main findings

Over the course of Time to Change (i.e. since 2008), our measures of experienced discrimination show a fall followed by an increase. When using the proportion of mental health service users experiencing any discrimination and the mean number of life areas in which discrimination is reported, there is no difference comparing 2008 and 2012. The overall median discrimination score is significantly less than in 2008, but nevertheless shows the same pattern of an increase following an initial fall. Concerning specific life areas in which experienced discrimination was assessed, significant increases were found comparing 2011–2012 for areas which had previously improved during Time to Change Phase 1, i.e. friends, social life and the experience of being shunned [7]. Regarding the total duration of 2008–2012, we found a significant increase with respect to welfare benefits. Consistent with these increases in experienced discrimination, we also found evidence of an increase in feeling the need to conceal one’s diagnosis between 2011 and 2012. Further, our previous finding [10] that access to social capital is inversely related to experienced discrimination was supported with respect to the skills subscale, in that a small reduction in the score accompanied an increase in experienced discrimination.

### Strengths and limitations

It is not known whether and if so how experienced and anticipated discrimination were changing before the baseline point in 2008 nor how contemporaneous socio-political factors may have contributed to these changes over time, so that neither the positive or negative changes can be directly attributed to the Time to Change programme. The other key limitation of this study is the low response rate. Following the rate of 6 % in 2008, two changes were made to the 2009–2010 recruitment strategy. Despite these changes, only 7 and 7.6 % of people who received an invitation pack were interviewed in 2009 and 2010, respectively. In 2011–2012, two further changes––an invitation letter from the participating Mental Health trust, and the offer of a £10 voucher for taking part in the survey––increased the response rate to 11 and 10 %, respectively.

There are a number of factors that may have affected the low response rate. First, participants had to respond to the initial mail out by sending back a consent form to the research team. After this, they would be called by an interviewer who would verbally confirm consent before starting the interview, creating a “two step” consent procedure. Second, recruitment relied on sampling through NHS trust patient databases. These databases may not have been accurate or up-to-date. Between 46 and 176 packs per year were returned as undeliverable to the trusts and it is likely that more were undelivered, but not returned. Third, the consistently low response rate may also reflect the nature of the population, many of whom may struggle to engage with a study of this kind due to their illness or relationship with mental health services. Finally, this population, especially participants from the London NHS trusts, may be asked to participate in research quite regularly and, therefore, may be experiencing research fatigue.

While we cannot determine the true degree or effect of any response bias, we were able to determine the extent to which the sample is representative of the entire population of non-institutionalised NHS mental health service users aged 18–65 with respect to age, ethnicity and gender. Comparison with these data shows that our sample under-represents younger people, non-white ethnic minorities and men, and that this was more the case in 2008 than in 2012. However, we found no association with TTC awareness and experienced discrimination for the 2009–2012 sample. It may be instead that as awareness of TTC rises, the group that are aware become more similar to those who are not with respect to their perception of unfair treatment.

The results in theory may have been affected by changes to simplify the wording of the survey instrument, as a revised version of the DISC was used from 2009. The main change was that ‘treated differently, and worse’ was replaced by ‘treated unfairly’ in each item on experienced discrimination. The changes lowered the Flesch–Kincaid reading grade to level 7.4 (i.e. understandable by the average US 7–8th grader) from 13.2 (i.e. understandable by the average US 13th grader). However, subsequent validation of the DISC shows that the two sets of wording used in 2008 vs. 2009 onwards elicit similar responses [18]. Further, while each question was re-worded in the same way, this did not result in the same pattern of change in endorsement across all items. Instead, the frequency of reporting increased for a few items and fell for the rest.

In spite of the low response rate our sampling design is an improvement over previous similar surveys in England, as the sample is drawn from those using NHS mental health services rather than from memberships of national mental health charities. Further, the high reported rates of experienced discrimination are consistent with surveys using the same instrument and different data collection methods yielding higher response rates. Both face-to-face surveys [1, 3] and a recent postal questionnaire to service users in New Zealand [26] have been conducted.

### Implications for research and policy

The pattern for the most commonly identified sources of negative discrimination, i.e. family and friends, as well as employers, of initial reductions being largely lost due to more recent increases is not consistent with changes in public attitudes to mental illness, which over 2009–2012 showed small, but positive changes [27]. Our results are more consistent with results from surveys on discrimination against people with physical disabilities [28] than with data on public attitudes to mental illness. One interpretation is that while public attitudes towards mental illness are improving, many people with mental illnesses are nevertheless adversely affected by increasingly negative attitudes and behaviour towards people who are, or who are assumed to be, in receipt of welfare benefits due to a disability [29]. Around half the Viewpoint sample describe themselves as unemployed, however, the existence of in-work benefits means that others in the sample may also be adversely affected by this trend. Thus, for the Viewpoint sample, any positive impact of the Time to Change Programme [30] may have been mitigated by a more recent negative trend in attitudes towards welfare recipients [29]. Public attitudes towards other minority groups may worsen during economic recessions as competition for employment increases [31]; this may also apply to mental illness. For example, a synthesis of public attitude trends in the US between the 1950s and 1990s showed improvements and declines which mirrored the economic and employment context of the country [32]. Another series of surveys suggests that the German public’s unwillingness to recommend an individual with depression for a job increased between 2000 and 2011 (i.e. following an economic recession) as compared to 1990–2000 [33]. Finally, European data [34] suggest that the gap in unemployment rates between individuals with and without mental health problems significantly widened during the recent economic recession, suggesting that the economic crisis had a greater impact on people with mental health problems; this was especially in countries with higher levels of stigma. It should be noted that while adjusting for employment status clarifies the effect of year of the survey, the increased level of unemployment among the 2011 and 2012 samples likely reflects a real increase in the population of those using mental health services due to the poor economic situation. Thus, one aspect of the effect of year is the increased unemployment rate, and adjusting for employment may thus represent an over-adjustment.

Some life areas show patterns across the 5 years other than an initial improvement followed by decline as described above. First, no significant change in reported discrimination from health professionals has been found. The literature suggests a number of reasons why mental health professionals’ behaviour may be more resistant to change: professional contact selects for people with the most severe course and outcome (the ‘physician’s bias’); contact occurs in the context of an unequal power relationship, and prejudice against the client group is one aspect of burnout, which is not uncommon among mental health professionals [35]. The extent to which reports of experienced discrimination are relevant to the provision of worse physical health care and higher mortality rates among people with mental illness is not known [36–39].

Second, in the area of welfare benefits a gradual increase in frequency of experienced discrimination has occurred (1) which is statistically significant for 2008–2012. This may be due to recent changes to the UK benefits system which mean that for many service users contact with the benefits system is more frequent and/or more aversive. The changes include the need for people on disability benefits to undergo an annual Work Capability Assessment, following which the entitlement is withdrawn if the claimant is found ‘fit for work’. This procedure has been widely criticised by health professionals [40] and politicians [41] for example on the basis that many people with disabilities have been wrongly found fit for work; such experiences have been reported by Viewpoint respondents. However, respondents’ examples also reflect more general problems, including the behaviour of staff administering welfare benefits and not receiving correct information on entitlements. The changes to the benefits system may have increased respondents’ exposure to such problems, either due to staff responses to the policy changes and/or simply due to increased frequency of contact with staff.

We have previously found that discrimination from friends and in finding a job are associated with reduced access to social capital [16], but the causal direction of the relationship between discrimination and access to social resources has yet to be elucidated. Experimental studies are required to explore the effect of interventions which increase access to social capital on discrimination. Last, it remains to be seen whether programmes such as Time to Change can impact on experiences in life areas affected by specific policies such as welfare benefits, especially during times of economic austerity and job insecurity. We have shown that experiences of discrimination can change in positive and negative directions; our results support the view that they may be influenced both by concerted national anti-stigma programmes and by the prevailing economic climate, and that these forces appear to act in opposite directions.
